# Supplementary material for: SAMM50-rs2073082, -rs738491 and -rs3761472 Interactions Enhancement of Susceptibility to Non-Alcoholic Fatty Liver Disease
Source: Biomedicines. 2023 Aug 29;11(9):2416. doi: 10.3390/biomedicines11092416 (PMC10525902; doi:10.3390/biomedicines11092416)
Supplement: Supplementary file 1 [file biomedicines-11-02416-s001.zip › biomedicines-2540428-supplementary.pdf]

Table S1. Multiple comparison correction of LSM based on Table 4

SNP	β	*SE*	*P*-value
rs2073082	-0.192	0.212	0.365
rs738491	0.304	0.139	0.029
rs3761472	0.355	0.131	0.007

Multiple linear regression model was adjusted for age, gender, and body mass index.

Table S2. Association of *SAMM50 rs3761472* G allele carriers and non-carriers with TC,LDL after logistic regression model adjusted for sex, age, BMI and lipid-lowering agent

Model 1	OR (95% CI)	*P*-value	Model 2	OR (95% CI)	*P*-value
Sex	1.190(0.894,1.584)	0.232	Sex	1.164(0.877,1.543)	0.292
Age	0.980(0.957,1.005)	0.115	Age	0.981(0.957,1.005)	0.120
BMI	0.968(0.931,1.008)	0.113	BMI	0.970(0.932,1.009)	0.131
ipid-lowering agent	0.869(0.664,1.138)	0.308	lipid-lowering agent	0.876(0.669,1.146)	0.333
TC	0.873(0.782,0.976)	0.017	LDL	0.871(0.772,0.982)	0.024

BMI, body mass index; TC, total cholesterol.

Table S3. Comparison of clinical characteristics according to *SAMM50* genotypes within the whole population

	rs2073082				*rs738491*				*rs3761472*			
	GG	AG	AA	*P*-value	CC	CT	TT	*P*-value	AA	AG	GG	*P*-value
N	441	497	115		325	521	207		415	515	123	
Age(year)	69.00 [67.00~74.00]	69.00 [67.00~73.00]	70.00 [67.00~75.00]	0.096	70.00 [67.00~75.00]	69.00 [67.00~73.00]	69.00 [67.00~74.00]	0.004	69.00 [67.00~74.00]	69.00 [67.00~74.00]	69.00 [67.00~73.00]	0.151
Male, n (%)	129 (29.25)	139 (27.97)	35 (30.43)	0.835	98 (30.15)	151 (28.98)	54 (26.09)	0.594	123 (29.64)	146 (28.35)	34 (27.64)	0.872
Weight(Kg)	65.93 ± 10.20	65.48 ± 10.28	65.82 ± 10.14	0.796	65.93 ± 10.51	65.66 ± 10.43	65.46 ± 9.27	0.869	66.34 ± 10.42	65.17 ± 10.20	65.78 ± 9.60	0.222
BMI(Kg/m^2)	25.80 ± 3.14	25.85 ± 3.26	25.70 ± 2.98	0.896	25.84 ± 3.27	25.85 ± 3.22	25.67 ± 2.94	0.769	25.98 ± 3.26	25.70 ± 3.17	25.73 ± 2.87	0.381
Systolic pressure	136.36 ± 19.70	134.06 ± 13.61	134.18 ± 13.97	0.090	135.80 ± 20.08	133.63 ± 13.78	137.34 ± 16.22	0.015	135.45 ± 19.27	134.13 ± 13.89	137.38 ± 16.01	0.120
Diastolic pressure	79.00 ± 9.56	78.87 ± 8.13	78.33 ± 7.79	0.768	78.87 ± 9.24	78.69 ± 8.44	79.30 ± 8.60	0.697	79.00 ± 9.28	78.60 ± 8.36	79.51 ± 8.23	0.546
Waist circumference	88.75 ± 7.99	88.78 ± 8.52	88.61 ± 8.10	0.979	89.09 ± 8.59	88.73 ± 8.38	88.25 ± 7.34	0.520	89.07 ± 8.51	88.56 ± 8.34	88.45 ± 6.91	0.600
Hip circumference	98.45 ± 7.36	98.40 ± 7.51	98.68 ± 7.17	0.938	98.58 ± 7.50	98.53 ± 7.51	98.04 ± 6.98	0.673	98.77 ± 7.52	98.22 ± 7.39	98.33 ± 7.03	0.518
WHR	0.90 ± 0.04	0.90 ±	0.90 ±	0.747	0.90 ±	0.90 ±	0.90 ±	0.560	0.90 ±	0.90 ±	0.90 ±	0.945

		0.05	0.05		0.06	0.05	0.04		0.05	0.05	0.04	
CAP	272.55 ± 49.16	275.01 ± 49.70	264.04 ± 47.78	0.156 (0.083)	268.10 ± 48.03	277.40 ± 50.36	268.84 ± 47.91	0.024	271.60 ± 47.75	275.01 ± 50.13	268.23 ± 51.14	0.373
LSM	5.22 ± 2.19	4.94 ± 1.72	4.85 ± 2.00	0.083	4.84 ± 1.68	5.09 ± 1.91	5.25 ± 2.39	0.080	4.85 ± 1.78	5.11 ± 1.84	5.42 ± 2.78	0.021
NFS	-0.68 [-1.40-0.12]	-0.79 [-1.48--0 .15]	-0.92 [-1.49-- 0.08]	0.135	-0.76 [-1.46-0. 09]	-0.74 [-1.44-0. 10]	-0.74 [-1.48-0. 04]	0.918	-0.73 [-1.44-0. 07]	-0.79 [-1.46--0.0 7]	-0.72 [-1.55-0. 03]	0.617
LSM≥8.2 Kpa, n (%)	29 (6.58)	24 (4.83)	4 (3.48)	0.273	12 (3.69)	29 (5.57)	16 (7.73)	0.133	18 (4.34)	26 (5.05)	13 (10.57)	0.024
NFS≥-1.455, n (%)	292 (66.21)	305 (61.37)	69 (60.00)	0.361	214 (65.85)	319 (61.23)	133 (64.25)	0.950	265 (63.86)	326 (63.30)	75 (60.98)	0.933
TBIL(umol/L)	15.97 ± 7.97	15.13 ± 5.77	14.98 ± 4.41	0.112	15.28 ± 5.66	15.33 ± 6.16	16.13 ± 8.99	0.281	15.32 ± 5.80	15.65 ± 7.35	15.21 ± 6.49	0.681
TG (mmol/L)	1.55 ± 1.08	1.63 ± 1.04	1.51 ± 0.92	0.395	1.56 ± 1.03	1.66 ± 1.12	1.44 ± 0.84	0.028	1.60 ± 1.08	1.60 ± 1.04	1.46 ± 0.94	0.394
TC (mmol/L)	4.74 ± 1.18	4.87 ± 1.29	4.89 ± 1.09	0.209	4.86 ± 1.15	4.84 ± 1.29	4.69 ± 1.19	0.260	4.92 ± 1.18	4.74 ± 1.27	4.80 ± 1.19	0.089
HDL (mmol/L)	1.16 ± 0.26	1.15 ± 0.24	1.18 ± 0.27	0.391	1.18 ± 0.26	1.14 ± 0.25	1.16 ± 0.23	0.121	1.16 ± 0.25	1.15 ± 0.25	1.18 ± 0.25	0.402
LDL (mmol/L)	3.27 ± 1.07	3.39 ± 1.18	3.41 ± 1.00	0.189	3.37 ± 1.05	3.37 ± 1.18	3.24 ± 1.06	0.360	3.44 ± 1.08	3.27 ± 1.16	3.33 ± 1.06	0.085
ALT(U/L)	19.83 ± 9.37	19.82 ± 9.27	19.03 ± 9.50	0.690	19.21 ± 8.73	20.11 ± 9.45	19.65 ± 9.94	0.385	19.52 ± 9.41	20.01 ± 9.41	19.38 ± 8.74	0.662
AST(U/L)	18.53 ± 7.76	18.52 ± 7.42	18.31 ± 6.67	0.961	18.21 ± 6.14	18.77 ± 7.74	18.28 ± 8.64	0.511	18.61 ± 7.26	18.37 ± 7.13	18.68 ± 9.43	0.856
Hypertension, n (%)	349 (79.14)	373 (75.05)	88 (76.52)	0.291	249 (76.62)	398 (76.39)	163 (78.74)	0.891	322 (77.59)	388 (75.34)	100 (81.30)	0.471
T2DM, n(%)	195 (44.22)	203 (40.85)	43 (37.39)	0.332	130 (40.00)	225 (43.19)	86 (41.55)	0.661	174 (41.93)	217 (42.14)	50 (40.65)	0.946
Stroke, n(%)	70 (15.87)	76 (15.29)	19 (16.52)	0.939	48 (14.77)	88 (16.89)	29 (14.01)	0.514	65 (15.66)	82 (15.92)	18 (14.63)	0.918
METS, n(%)	376 (85.26)	422 (84.91)	95 (82.61)	0.777	272 (83.69)	446 (85.60)	175 (84.54)	0.748	354 (85.30)	434 (84.27)	105 (85.37)	0.895
Lipid lowering agent, n (%)	160 (36.28)	158 (31.79)	37 (32.17)	0.308	113 (34.77)	167 (32.05)	75 (36.23)	0.540	145 (34.94)	171 (33.20)	39 (3171)	0.828

BMI, body mass index; WHR, Waist-to-hip ratio; CAP, controlled attenuated parameter controlled; LSM, liver stiffness measurement; NFS, NAFLD Fibrosis Score; TBIL,total bilirubin; TG, total triglyceride; TC, total cholesterol; HDL, high-density lipoprotein; LDL, low-density lipoprotein; ALT, alanine aminotransferase; AST, aspartate aminotransferase; T2DM, type 2 diabetes; Mets, metabolic syndrome.

Table S4. Multiple comparison correction of LSM based on Table S3

SNP	β	*SE*	*P*-value
rs2073082	-0.226	0.099	0.022
rs738491	0.219	0.091	0.016
rs3761472	0.298	0.097	0.002

Multiple linear regression model was adjusted for age, gender, and body mass index.

Table S5. Comparison of clinical characteristics according to SAMM50 genotypes within NAFLD cohorts

	rs2073082				*rs738491*				*rs3761472*			
	GG	AG	AA	*P*-value	CC	CT	TT	*P*-value	AA	AG	GG	*P*-value
N	242	299	49		161	312	117		230	290	70	
Age(year)	69.00 [66.00~73.00]	68.00 [67.00~72.00]	70.00 [66.50~74.50]	0.497	70.00 [67.00~74.00]	68.00 [67.00~72.00]	69.00 [66.00~72.00]	0.037	69.00 [67.00~74.00]	68.00 [66.00~73.00]	69.00 [66.00~71.00]	0.077
Male, n (%)	63 (26.03)	69 (23.08)	17 (34.69)	0.208	44 (27.33)	80 (25.64)	25 (21.37)	0.515	60 (26.09)	72 (24.83)	17 (24.29)	0.929
Weight(Kg)	67.76 ± 10.67	68.43 ± 10.01	70.27 ± 10.38	0.288	69.11 ± 10.18	68.23 ± 10.62	67.41 ± 9.69	0.389	69.22 ± 10.23	67.71 ± 10.44	67.78 ± 10.06	0.228
BMI(Kg/m^2)	26.68 ± 3.03	27.01 ± 3.16	27.22 ± 2.53	0.329	27.05 ± 2.98	26.93 ± 3.18	26.55 ± 2.84	0.372	27.12 ± 3.04	26.79 ± 3.11	26.53 ± 2.86	0.272
Systolic pressure	137.54 ± 22.36	135.39 ± 13.73	134.75 ± 13.22	0.320	137.58 ± 24.70	134.61 ± 13.31	138.57 ± 16.16	0.064	137.20 ± 22.37	134.90 ± 13.23	138.32 ± 16.15	0.200
Diastolic pressure	79.62 ± 10.49	79.39 ± 8.33	79.10 ± 7.71	0.923	78.62 ± 10.59	79.61 ± 8.60	80.22 ± 8.71	0.335	79.28 ± 10.40	79.42 ± 8.40	80.20 ± 8.19	0.763
Waist circumference	90.77 ± 7.62	91.25 ± 8.13	91.98 ± 7.94	0.575	91.99 ± 8.19	90.97 ± 8.03	90.31 ± 7.10	0.198	91.79 ± 8.08	90.73 ± 8.04	90.46 ± 6.62	0.242
Hip circumference	99.83 ± 7.29	100.73 ± 7.14	100.35 ± 6.58	0.356	100.65 ± 7.00	100.42 ± 7.34	99.67 ± 6.89	0.513	100.84 ± 7.18	100.10 ± 7.16	99.58 ± 7.05	0.329
WHR	0.91 ± 0.04	0.91 ± 0.05	0.92 ± 0.05	0.329	0.91 ± 0.05	0.91 ± 0.05	0.91 ± 0.04	0.231	0.91 ± 0.05	0.91 ± 0.05	0.91 ± 0.04	0.692
CAP	293.87 ± 41.67	296.52 ± 40.14	293.49 ± 45.30	0.758	291.94 ± 43.67	299.00 ± 39.28	289.67 ± 41.68	0.080	294.42 ± 40.99	296.59 ± 40.02	292.11 ± 46.15	0.705
LSM	5.57 ± 2.16	5.31 ± 1.76	5.64 ± 2.48	0.306	5.31 ± 1.87	5.41 ± 1.80	5.70 ± 2.54	0.318	5.33 ± 1.94	5.43 ± 1.76	5.88 ± 2.86	0.162
NFS	-0.69 [-1.42-0.10]	-0.74 [-1.37--0.20]	-0.97 [-1.48-0.00]	0.756	-0.75 [-1.39-0.23]	-0.69 [-1.38--0.14]	-0.75 [-1.55--0.06]	0.727	-0.71 [-1.38-0.03]	-0.78 [-1.42--0.20]	-0.72 [-1.55-0.10]	0.311
LSM≥8.2 Kpa, n (%)	20 (8.26)	20 (6.69)	3 (6.12)	0.694	10 (6.21)	21 (6.73)	12 (10.26)	0.415	16 (6.96)	18 (6.21)	9 (12.86)	0.154
NFS≥-1.455	148 (61.16)	180 (60.20)	26 (53.06)	0.457	105 (65.22)	183 (58.65)	66 (56.41)	0.372	142 (61.74)	173 (59.66)	39 (55.71)	0.766

TBIL(umol/L)	16.38 ± 9.41	15.08 ± 5.83	15.51 ± 4.93	0.133	15.43 ± 6.41	15.30 ± 6.06	16.88 ± 11.24	0.136	15.42 ± 6.63	15.90 ± 8.23	15.36 ± 6.81	0.728
TG (mmol/L)	1.78 ± 1.25	1.78 ± 1.09	1.78 ± 1.19	0.996	1.78 ± 1.18	1.85 ± 1.22	1.60 ± 0.97	0.150	1.81 ± 1.22	1.79 ± 1.13	1.67 ± 1.14	0.687
TC (mmol/L)	4.65 ± 1.14	4.95 ± 1.37	4.85 ± 1.22	0.024	4.85 ± 1.20	4.86 ± 1.35	4.65 ± 1.16	0.306	4.90 ± 1.22	4.77 ± 1.32	4.73 ± 1.24	0.424
HDL (mmol/L)	1.11 ± 0.23	1.13 ± 0.21	1.10 ± 0.25	0.456	1.13 ± 0.24	1.10 ± 0.22	1.13 ± 0.21	0.228	1.11 ± 0.22	1.12 ± 0.23	1.13 ± 0.22	0.803
LDL (mmol/L)	3.18 ± 1.00	3.46 ± 1.27	3.40 ± 1.09	0.019	3.36 ± 1.07	3.39 ± 1.24	3.20 ± 1.03	0.329	3.43 ± 1.09	3.29 ± 1.22	3.26 ± 1.09	0.363
ALT(U/L)	21.45 ± 10.51	21.24 ± 9.45	23.04 ± 12.21	0.514	21.11 ± 9.67	21.58 ± 9.92	21.71 ± 11.36	0.859	21.22 ± 10.38	21.46 ± 10.09	22.36 ± 9.67	0.716
AST(U/L)	19.33 ± 8.90	19.19 ± 8.30	20.55 ± 8.68	0.587	19.12 ± 7.19	19.37 ± 8.48	19.68 ± 10.43	0.867	19.46 ± 8.40	18.96 ± 7.85	20.70 ± 11.52	0.305
Hypertension, n (%)	193 (79.75)	230 (76.92)	42 (85.71)	0.278	128 (79.50)	246 (78.85)	91 (77.78)	0.849	187 (81.30)	219 (75.52)	59 (84.29)	0.240
T2DM, n(%)	116 (47.93)	135 (45.15)	21 (42.85)	0.725	73 (45.34)	146 (46.79)	53 (45.30)	0.938	109 (47.39)	129 (44.48)	34 (48.57)	0.729
Stroke, n(%)	39 (16.12)	47 (15.72)	6 (12.24)	0.790	23 (14.29)	53 (16.99)	16 (13.68)	0.578	36 (15.65)	45 (15.52)	11 (15.71)	0.999
METS, n(%)	218 (90.08)	275 (91.97)	44 (89.80)	0.711	143 (88.82)	292 (93.59)	102 (87.18)	0.061	212 (92.17)	263 (90.69)	62 (88.57)	0.629
Lipid lowering agent, n (%)	78 (32.23)	96 (32.11)	17 (34.69)	0.931	54 (33.54)	100 (32.05)	37 (31.62)	0.936	80 (34.78)	89 (30.69)	22 (31.43)	0.722

BMI, body mass index; WHR, Waist-to-hip ratio; CAP, controlled attenuated parameter controlled; LSM, liver stiffness measurement; NFS, NAFLD Fibrosis Score; TBIL,total bilirubin; TG, total triglyceride; TC, total cholesterol; HDL, high-density lipoprotein; LDL, low-density lipoprotein; ALT, alanine aminotransferase; AST, aspartate aminotransferase; T2DM, type 2 diabetes; Mets, metabolic syndrome.

Table S6. Comparison of clinical characteristics according to SAMM50 genotypes within NAFLD cohorts（Carriers Vs Noncarriers）

	rs2073082			*rs738491*			*rs3761472*		
	Carriers(GG+AG)	*Noncarriers(AA)*	*P*-value	*Carriers(TT+CT)*	*Noncarriers(CC)*	P-value	*Carriers(GG+AG)*	*Noncarriers(AA)*	*P*-value
N	541	49		429	161		360	230	
Age(year)	69.00 [66.00~73.00]	69.00 [67.00~73.00]	0.316	68.00 [66.00~72.00]	70.00 [67.00~74.00]	0.010	68.00 [66.00~72.00]	69.00 [67.00~74.00]	0.026
Male, n (%)	132 (24.40)	17 (34.69)	0.112	105 (24.48)	44 (27.33)	0.477	89 (24.72)	60 (26.09)	0.710
Weight(Kg)	68.13 ± 10.31	70.27 ± 10.38	0.165	68.00 ± 10.37	69.11 ± 10.18	0.245	67.72 ± 10.35	69.22 ± 10.23	0.086
BMI(Kg/m2)	26.86 ± 3.10	27.22 ± 2.53	0.434	26.83 ± 3.09	27.05 ± 2.98	0.425	26.74 ± 3.06	27.12 ± 3.04	0.137
Systolic pressure	136.35 ± 18.09	134.75 ± 13.22	0.550	135.70 ± 14.25	137.58 ± 24.70	0.256	135.57 ± 13.89	137.20 ± 22.37	0.282
Diastolic pressure	79.49 ± 9.34	79.10 ± 7.71	0.782	79.78 ± 8.63	78.62 ± 10.59	0.177	79.57 ± 8.36	79.28 ± 10.40	0.707
Waist	91.04 ± 7.91	91.98 ± 7.94	0.431	90.79 ± 7.78	91.99 ± 8.19	0.103	90.68 ± 7.77	91.79 ± 8.08	0.096

circumference									
Hip circumference	100.33 ± 7.21	100.35 ± 6.58	0.981	100.21 ± 7.22	100.65 ± 7.00	0.513	100.00 ± 7.13	100.84 ± 7.18	0.164
WHR	0.91 ± 0.05	0.92 ± 0.05	0.212	0.91 ± 0.05	0.91 ± 0.05	0.087	0.91 ± 0.05	0.91 ± 0.05	0.456
LSM	5.43 ± 1.95	5.64 ± 2.48	0.526	5.49 ± 2.03	5.31 ± 1.87	0.367	5.52 ± 2.02	5.33 ± 1.94	0.306
CAP	295.37 ± 40.79	293.49 ± 45.30	0.784	296.43 ± 40.12	291.94 ± 43.67	0.275	295.72 ± 41.24	294.42 ± 40.99	0.729
NFS	-0.73 [-1.40--0.06]	-0.97 [-1.48-0.00]	0.814	-0.72 [-1.41--0.13]	-0.75 [-1.39-0.23]	0.476	-0.75 [-1.43--0.12]	-0.71 [-1.38-0.03]	0.209
LSM≥8.2 Kpa, n (%)	40 (7.39)	3 (6.12)	0.863	33 (7.70)	10 (6.21)	0.577	27 (7.50)	16 (6.96)	0.863
NFS≥-1.455	328 (60.63)	26 (53.06)	0.355	249 (58.04)	105 (65.22)	0.538	212 (58.89)	142 (61.74)	0.523
TBIL(umol/L)	15.66 ± 7.66	15.51 ± 4.93	0.888	15.73 ± 7.84	15.43 ± 6.41	0.668	15.79 ± 7.97	15.42 ± 6.63	0.556
TG (mmol/L)	1.78 ± 1.16	1.78 ± 1.19	0.983	1.78 ± 1.16	1.78 ± 1.18	0.977	1.76 ± 1.13	1.81 ± 1.22	0.670
TC (mmol/L)	4.81 ± 1.28	4.85 ± 1.22	0.828	4.80 ± 1.30	4.85 ± 1.20	0.692	4.76 ± 1.30	4.90 ± 1.22	0.197
HDL (mmol/L)	1.12 ± 0.22	1.10 ± 0.25	0.502	1.11 ± 0.22	1.13 ± 0.24	0.260	1.12 ± 0.22	1.11 ± 0.22	0.757
LDL (mmol/L)	3.34 ± 1.16	3.40 ± 1.09	0.710	3.33 ± 1.19	3.36 ± 1.07	0.811	3.29 ± 1.19	3.43 ± 1.09	0.158
ALT(U/L)	21.33 ± 9.94	23.04 ± 12.21	0.259	21.61 ± 10.32	21.11 ± 9.67	0.591	21.63 ± 10.00	21.22 ± 10.38	0.634
AST(U/L)	19.25 ± 8.56	20.55 ± 8.68	0.311	19.45 ± 9.04	19.12 ± 7.19	0.674	19.30 ± 8.69	19.46 ± 8.40	0.817
Hypertension, n (%)	423 (78.19)	42 (85.71)	0.191	337 (78.55)	128 (79.50)	0.795	278 (797.22)	187 (81.30)	0.405
T2DM, n(%)	251 (46.40)	21 (42.86)	0.634	199 (46.39)	73 (45.34)	0.821	163 (45.27)	109 (47.39)	0.615
Stroke, n(%)	86 (15.90)	6 (12.24)	0.502	69 (16.08)	23 (14.29)	0.591	56 (15.56)	36 (15.65)	0.958
METS, n(%)	493 (91.13)	44 (89.80)	0.755	394 (91.84)	143 (88.82)	0.253	325 (90.28)	212 (92.17)	0.432
Lipid lowering agent, n (%)	174 (32.16)	17 (34.69)	0.709	137 (31.93)	54 (33.54)	0.759	111 (30.83)	80 (34.78)	0.420

BMI, body mass index; WHR, Waist-to-hip ratio; CAP, controlled attenuated parameter controlled; LSM, liver stiffness measurement; NFS, NAFLD Fibrosis Score; TBIL,total bilirubin; TG, total triglyceride; TC, total cholesterol; HDL, high-density lipoprotein; LDL, low-density lipoprotein; ALT, alanine aminotransferase; AST, aspartate aminotransferase; T2DM, type 2 diabetes; Mets, metabolic syndrome.
